# Mutational screening of six genes in Chinese patients with congenital cataract and microcornea

**Published:** 2011-06-07

**Authors:** Wenmin Sun, Xueshan Xiao, Shiqiang Li, Xiangming Guo, Qingjiong Zhang

**Affiliations:** State Key Laboratory of Ophthalmology, Zhongshan Ophthalmic Center, Sun Yat-sen University, Guangzhou, China

## Abstract

**Purpose:**

To identify mutations in 6 genes of 9 Chinese families with congenital cataract and microcornea.

**Methods:**

Nine unrelated families with congenital cataract and microcornea were collected. Cycle sequencing was used to detect variants in the coding and adjacent regions of the crystallin alpha A (*CRYAA*), crystallin beta B1 (*CRYBB1*), crystallin beta A4 (*CRYBA4*), crystallin gamma C (*CRYGC*), crystallin gamma D (*CRYGD*), and gap junction protein alpha 8 (*GJA8*) genes.

**Results:**

Upon complete analysis of the 6 genes, three mutations in 2 genes were detected in 3 families, respectively. These mutations were not present in 96 normal controls. Of the three mutations, two novel heterozygous mutations in *GJA8*, c.136G>A (p.Gly46Arg) and c.116C>G (p.Thr39Arg), were found in two families with congenital cataract and microcornea. The rest one, a heterozygous c.34C>T (p.Arg12Cys) mutation in *CRYAA*, was identified in three patients from a family with nuclear cataract, microcornea with axial elongation. No mutation in the 6 genes was detected in the remaining 6 families.

**Conclusions:**

Mutations in *GJA8* and *CRYAA* were identified in three families with cataract and microcornea. Elongation of axial length accompanied with myopia was a novel phenotype in the family with the c.34C>T mutation in *CRYAA*. Our results expand the spectrum of *GJA8* mutations as well as their associated phenotypes.

## Introduction

Congenital cataract is a leading cause of childhood blindness accounting for about 10%~38% of blindness in children [1], with a prevalence around 0.006%~0.06% in live births [2,3]. It may occur alone or associated with other ocular or systemic abnormalities. Microcornea, one of the most frequent abnormalities associated with congenital cataract, results from the secondary damage of the lens maldevelopment or from mutations in some growth or transcription factors [4]. To date, around 200 genes and loci have been associated with cataracts [4,5]. Of these genes, mutations in at least 9 genes were reported to be responsible for congenital cataract associated with microcornea, including genes encoding crystallins (crystallin alpha-A [*CRYAA*], OMIM 123580; crystallin beta-A4 [*CRYBA4*], OMIM 123631; crystallin beta-B1 [*CRYBB1*], OMIM 600929; crystallin beta-B2 [*CRYBB2*], OMIM 123620; crystallin gamma-C [*CRYGC*], OMIM 123680; and crystallin gamma-D [*CRYGD*], OMIM 123690) [6-14], gap junction protein alpha 8 (*GJA8*, OMIM 600897) [6,15], v-maf avian musculoaponeurotic fibrosarcoma oncogene homolog (*MAF*, OMIM 177075) [16,17], and solute carrier family 16 member 12 (*SLC16A12*, OMIM 611910) [18]. Analyses of individual gene in patients with cataract and microcornea have been frequently reported [8-16,18-21] but comprehensive analysis of all these genes in the same set of families is rare [6].

In this study, we performed mutational screening of 6 genes (*CRYAA*, *CRYBB1*, *CRYBA4*, *CRYGC*, *CRYGD*, and *GJA8*) in 9 Chinese families with congenital cataract and microcornea. Three mutations in *GJA8* and *CRYAA* were identified in 3 families.

## Methods

### Patients

Nine families with congenital cataract and microcornea were collected at the Pediatric and Genetic Eye Clinic of the Zhongshan Ophthalmic Center, Guangzhou, China. Written informed consent conforming to the tenets of the Declaration of Helsinki and following the Guidance of Sample Collection of Human Genetic Diseases (863-plan) by the Ministry of Public Health of China were obtained from the participating individuals or their guardians before the study. Congenital cataract represents cataract presented at birth or noticed in the first few months after birth. Microcornea represents a cornea with horizontal diameter of less than 10 mm. Genomic DNA was prepared from leukocytes of peripheral venous blood using the standard phenol/chloroform method [22].

### Mutation detection

Genomic bioinformation of the 6 genes was obtained from the National Center for Biotechnology Information (NCBI): *CRYAA* (NCBI human genome build 37.2, NC_000021.8 for gDNA, NM_000394.2 for mRNA and NP_000385.1 for protein), CRYBB1 (NCBI human genome build 37.2, NC_000022.10 for gDNA, NM_001887.3 for mRNA and NP_001878.1 for protein), CRYBA4 (NCBI human genome build 37.2, NC_000022.10 for gDNA, NM_001886.2 for mRNA and NP_001877.1 for protein), CRYGC (NCBI human genome build 37.2, NC_000002.11 for gDNA, NM_020989.3 for mRNA and NP_066269.1 for protein), CRYGD (NCBI human genome build 37.2, NC_000002.11 for gDNA, NM_006891.3 for mRNA and NP_008822.2 for protein), and GJA8 (NCBI human genome build 37.2, NC_000001.10 for gDNA, NM_005267.4 for mRNA and NP_005258.2 for protein). Primers used to amplify the coding exons and adjacent intronic regions of the 6 genes were referred to a previous publication [23] with modification for a few primers (Table 1). Individual exon was amplified by polymerase chain reaction (PCR). The sequence of the amplicons was determined with the ABI BigDye Terminator cycle sequencing kit v3.1 on a genetic analyzer (ABI Applied Biosystems, Foster City, CA). Sequencing results from patients were aligned with consensus sequences to identify variations by using the SeqManII program of the Lasergene package (DNAStar Inc., Madison, WI). A variant detected in patient was further evaluated in controls by sequencing 96 normal individuals.

**Table 1:** Primers used to amplify the coding and adjacent regions of the 6 genes.

**Gene**	**Primer name**	**Primer sequence (5′→3′)**	**Product length (bp)**	**Annealing temperature (°C)**
*CRYAA*	1F	GCTGGGGGCGGGCACTTG	552	68
	1R	TGGGGACACAGGCTCTCG		
	2F	GGTGACCGAAGCATCTCTGT	295	68
	2R	CGTGACCCCCTTGTCCTC		
	3F	ACCCGGCCCCTGTGAGAG	438	59
	3R	AAAGGGAAGCAAAGGAAGACA		
*CRYGC*	1–2F	CCAAATAAAAGCAACACAGAGC	671	63.8
	1–2R	AAACCTCCCTCCCTGTAACC		
	3F	CGCAGCAACCACAGTAATCT	579	59.2
	3R	CCCACCCCATTCACTTCTTA		
*CRYGD*	1–2F	GGGCCCCTTTTGTGCGGTTCT	643	65
	1–2R	GTGGGGAGCAAACTCTATTGA		
	3F	TGCTCGGTAATGAGGAGTTT	506	63
	3R	AAATCAGTGCCAGGAACACA		
*GJA8*	1aF	CAGATATTGACTCAGGGTTGC	475	60
	1aR	CCGCTGCTCTTCTTGACG		
	1bF	ATTCGCCTCTGGGTGCTG	571	58
	1bR	CCTTGGCTTTCTGGATGG		
	1cF	GCAGCAAAGGCACTAAGAA	578	60
	1cR	CACCTGAGCGTAGGAAGG		
	1dF	ATCGTTTCCCACTATTTCC	559	56
	1dR	GATCATGTTGGCACCTTTT		
*CRYBB1*	1F	GGTAATGGAGGGTGGAAC		
	1R	GAGAATAGGGACAGAGGATAAG	672	62
	2F	GGAGGACAGGATCATTTCA		
	2R	ATAATGTATGTGCCAGGAGTA	387	62
	3F	CCTTTGGACTTTCCTACTG		
	3R	GCTTTTGTGCTTATCATTT	483	58
	4F	TAGACAGCAGTGGTCCCT		
	4R	TTGATTACTCCTTCAACCC	571	60
	5F	TAGCCAGGACAGAAGTGAGA		
	5R	ATTGAACATGAAGAAGGGTT	362	60
*CRYBA4*	1F	CCCTAGCCCAGTCACTCCT		
	1R	TGAGCCTTGATTGCACCTCT	289	60
	2F	GGCACCTGTGCTGTCTAGTG		
	2R	GCCTAGGGAGAGGGGACCTA	396	62
	3F	CTCCCCTAGTCGTGACAACC		
	3R	TTTCAACTCTGGAACCTTTGA	394	62
	4F	TTATTGCCCTTCCAAAAGGTT		
	4R	TGTTCTCCTCTGGAATGTGG	397	62
	5F	AAAAGAAAGGCTGGGATGGT		
	5R	AAAACCGGTTCTTTGAAAAGATTA	584	62

### Variations analysis through online tools

The effects of alterations were evaluated by Polymorphism Phenotyping (PolyPhen-2) [24,25] and Sorting Intolerant From Tolerant (SIFT) [26] at the protein level.

## Results

**Figure 1:**
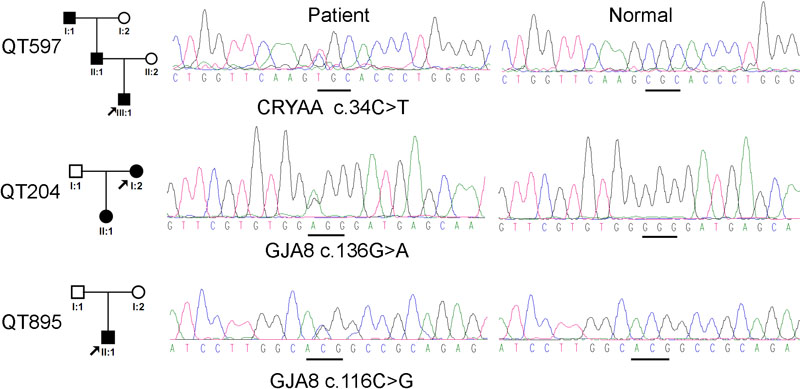
Mutations identified in 3 unrelated families with congenital cataract and microcornea. Pedigrees are shown in the left column. Sequence chromatography with mutation in each family is shown in the middle and the sequences from normal controls are aligned on the right column. Mutations in the 3 families were described under each sequence followed the nomenclature recommended by Human Genome Variation Society (HGVS).

**Figure 2:**
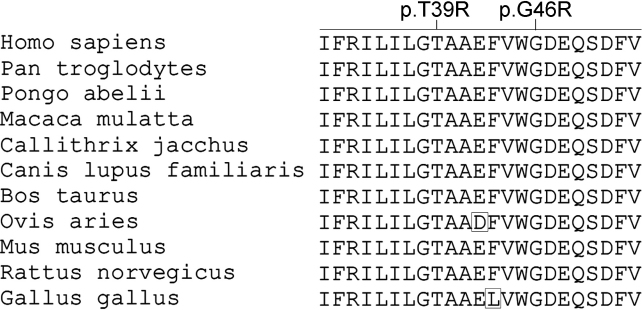
Protein sequence alignment of eleven GJA8 orthologs. The regions with the novel p.T39R and p.G46R mutations are highly conserved in the eleven species.

Upon complete analysis of the 6 genes, three heterozygous mutations in 2 genes were detected in 3 families (Figure 1), including c.34C>T (p.Arg12Cys) mutation in *CRYAA*, and c.116C>G (p.Thr39Arg) and c.136G>A (p.Gly46Arg) mutations in *GJA8*, where the last two mutations are novel. Both of the c.116C>G and c.136G>A mutations in *GJA8* are predicted to be “probably damaging” by PolyPhen-2 and “intolerant” by SIFT. The p.Thr39Arg would change the Blosum62 score from 4 to −1 whereas the p.Gly46Arg would change the Blosum62 score from 6 to −2. The p.Thr39Arg and p.Gly46Arg variants involved residues that are conserved across different species (Figure 2).

**Figure 3:**
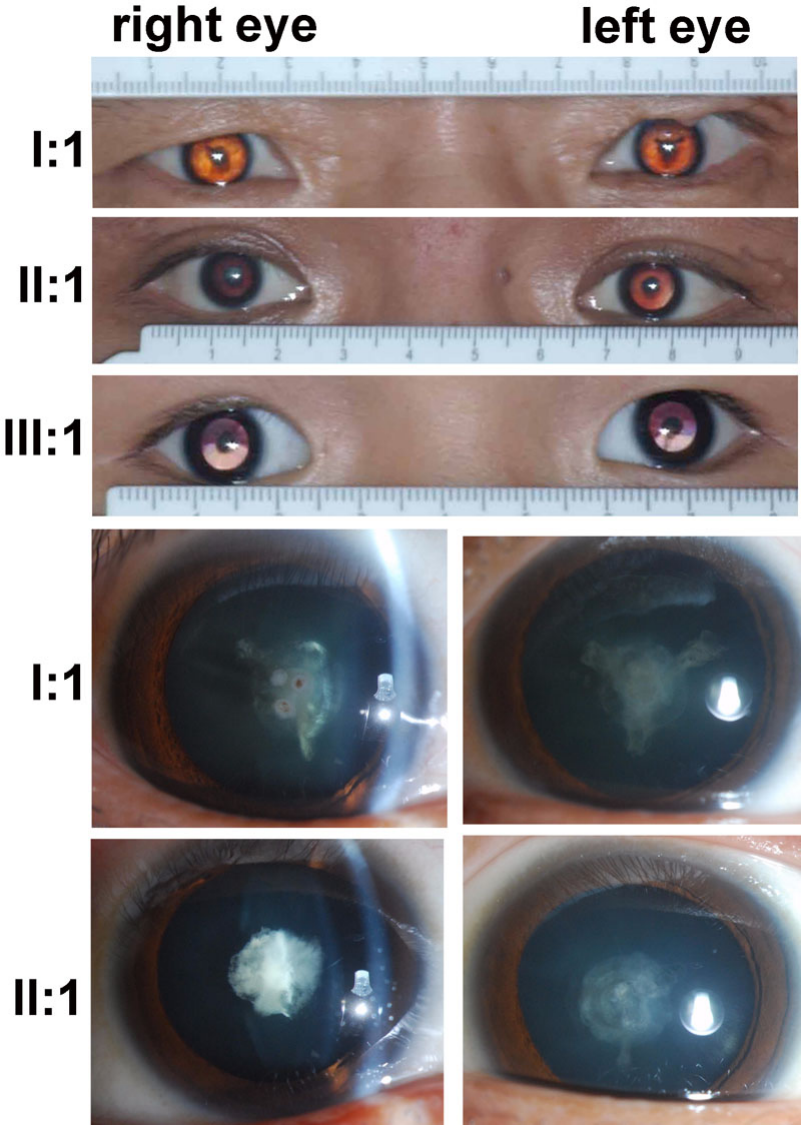
Photos shows the microcornea and nuclear cataracts of the three affected patients with a c.34C>T mutation in *CRYAA* in Family QT597. I:1, II:1, and III:1 on the left is the individual identification numbers that are the same as in the pedigree for QT597 in Figure 1. The top three photos demonstrated bilateral microcornea and bilateral nuclear cataracts in the three patients. The bottom two rows show the nuclear cataracts with suture opacity in I:1 and shell-like opacity in II:1.

The heterozygous c.34C>T mutation in *CRYAA* was identified in all three patients in a three-generation family (QT597), where all patients had congenital nuclear cataract and microcornea (Figure 3, Table 2). Myopic fundus change in both eyes were observed in the affected father (II:1) and affected grandfather (I:1; Figure 4). Ocular ultrasound recorded axial lengths of 24.47 mm for the right eye and 24.61 mm for the left eye of II:1 and that of 27.82 mm for the right eye and 26.35 mm for the left eye of I:1. The proband had −3.00D for both eyes at the age of 4 years old.

**Table 2:** Listed below is the clinical information of the patients with mutations.

**ID**	**Mutation**	**Gender**	**Age (years) at**		**Inheritance**	**Visual acuity (right;left)**	**Cataract types**	**Cornea size (right;left; mm)**	**Axial length (mm) (right;left)**
			**exam**	**onset**					
QT597I:1	c.34C>T; *CRYAA*	male	47	at birth	AD	0.04; 0.04	nuclear	10; 10	27.82; 26.35
QT597II:1	c.34C>T; *CRYAA*	male	24	at birth	AD	0.04; 0.08	nuclear	10; 10	24.47; 24.16
QT597III:1	c.34C>T; *CRYAA*	male	4	at birth	AD	N/A	nuclear	9.5; 9.5	N/A
QT204I:2	c.136G>A; *GJA8*	female	34	at birth	AD	NLP; 0.03	total	9; 9	N/A
QT204II:1	c.136G>A; *GJA8*	female	5	at birth	AD	0.2; 0.25	total	7; 7	N/A
QT895	c.116C>G; *GJA8*	male	7	at birth	Sporadic	0.05; 0.1	total	6; 6	N/A

**Figure 4:**
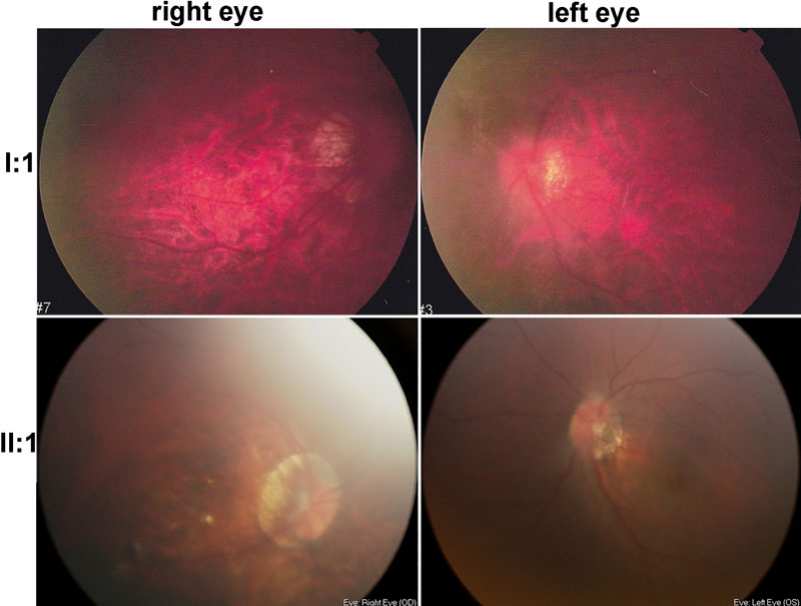
Fundus photos demonstrate obvious crescent choroidal defects in the temporal region of the optic disc. Tigroid retinal changes are present in posterior fundus.

The c.136G>A mutation in *GJA8* was identified in a two-generation family (QT204) with complete opacity of the lens and microcornea (Table 2). Horizontal cornea diameter was 9 mm for both eyes of the affected mother and 7 mm for both eyes of the affected daughter at the age of 5 years old.

**Figure 5:**
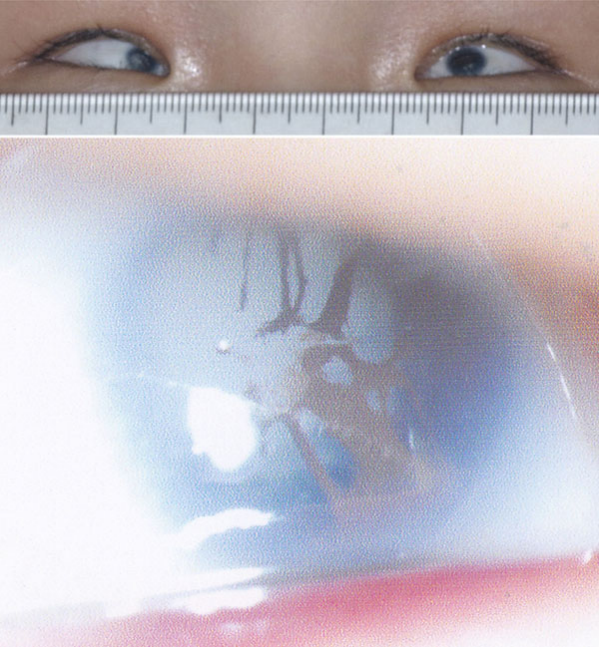
Microcornea and congenital cataracts observed in patient QT895. The top photo shows the small corneas and the bottom photo shows complete opacity of the lens and iris hypoplasia.

The c.116C>G mutation in *GJA8* was identified in a sporadic patient (QT895) of 7 years old with microcornea, complete opacity of lenses, and iris hypoplasia (Figure 5, Table 2). Horizontal corneal diameter was about 6 mm for both eyes.

## Discussion

In this study, we screened 6 genes for mutations in 9 Chinese families with congenital cataract and microcornea. Three mutations were identified in 3 of the 9 (30%) families, including a c.34C>T (p.Arg12Cys) in *CRYAA*, and a c.136G>A (p.Gly46Arg) and a c.116C>G (p.Thr39Arg) in *GJA8*, respectively.

*CRYAA* is located in 21q22.3 and encodes the α-A-crystallin in lens epithelial cells and fiber cells. α-A-crystallin is a member of small heat shock proteins with the chaperone activity which contributes to keeping lens transparent [6,10,27]. Up to now, there were eight mutations of *CRYAA* found in sixteen families most of which involved substitutions from or to arginine [5]. And the corresponding phenotypes of the mutations were related with congenital cataract with or without microcornea, microphthalmia, or iris coloboma.

We found a known c.34C>T (p.Arg12Cys) mutation in *CRYAA* of three patients from a family with congenital nuclear cataract and microcornea. Previously, this mutation has been identified in four families with nuclear or lamellar cataracts, and some patients accompanied with microcornea or microphthalmia [6,10,28,29]. Elongation of axial length or myopia has not been observed in previous studies.

*GJA8* is located in chromosome 1q21.1 and encodes the gap junction proteins, connexin50. *GJA8* is one of the most common genes causing congenital cataract with or without other ocular abnormalities. Previous studies showed that *GJA8*-knockout mice developed nuclear cataract and microphthalmia, from which it is considered that *GJA8* plays a role not only in keeping lens transparent but in ocular growth [30,31]. Up to now, about twenty mutations in *GJA8* have been associated with congenital cataracts in at least 21 families. Of these mutations, five were identified in five families with microcornea and two families accompanied with microphthamia [32,33].

In this study, we found two novel missense mutations c.136G>A and c.116C>G in *GJA8* in two families with congenital cataract and microcornea. The c.136G>A mutation led to a substitution from glycine to arginine at the amino acid position 46, and the c.116C>G mutation led to a substitution from threonine to arginine at the amino acid position 39. Both the 46 and 39 positions are located in the first transmembrane domain. In a previous study, Minogue et al. [32] identified a c.137G>T (p.Gly46Val) mutation in *GJA8* of a proband with early-onset total cataract accompanied with small eyes and pupils. Therefore, the three mutations may result in phenotype by the similar mechanism.

In summary, a known c.34C>T mutation in *CRYAA* and two novel mutation in *GJA8* were identified in 3 of 9 families after comprehensive analysis of 6 genes known to cause cataract and microcornea. Our results expand the mutation spectrum of *GJA8* and phenotypic variations associated with *CRYAA* mutations. Patients without mutation in the 6 genes are potential candidate for future study of additional causative genes for cataracts and microcornea.
